# An Exploratory Study on Resting-State Functional Connectivity in Individuals with Disorganized Attachment: Evidence for Key Regions in Amygdala and Hippocampus

**DOI:** 10.3390/brainsci11111539

**Published:** 2021-11-19

**Authors:** Gianluca Cruciani, Maddalena Boccia, Vittorio Lingiardi, Guido Giovanardi, Pietro Zingaretti, Grazia Fernanda Spitoni

**Affiliations:** 1 Department of Psychology, Ph.D. Program in Behavioral Neuroscience, Sapienza University of Rome, Via dei Marsi 78, 00185 Rome, Italy; 2 Department of Psychology, Sapienza University of Rome, Via dei Marsi 78, 00185 Rome, Italy; maddalena.boccia@uniroma1.it; 3 Cognitive and Motor Rehabilitation and Neuroimaging Unit, Santa Lucia Foundation (IRCCS Fondazione Santa Lucia), 00179 Rome, Italy; grazia.spitoni@uniroma1.it; 4 Department of Dynamic and Clinical Psychology, and Health Studies, Sapienza University of Rome, Via degli Apuli 1, 00185 Rome, Italy; vittorio.lingiardi@uniroma1.it (V.L.); guido.giovanardi@uniroma1.it (G.G.); 5 Villa von Siebenthal Neuropsychiatric Clinic and Hospital, Genzano di Roma, 00045 Rome, Italy; pie.zingaretti@gmail.com

**Keywords:** fMRI, organized attachment, unresolved attachment, adult attachment, resting-state functional connectivity

## Abstract

Studies comparing organized (O) and unresolved/disorganized (UD) attachment have consistently shown structural and functional brain abnormalities, although whether and how attachment patterns may affect resting state functional connectivity (RSFC) is still little characterized. Here, we investigated RSFC of temporal and limbic regions of interest for UD attachment. Participants’ attachment was classified via the Adult Attachment Interview, and all participants underwent clinical assessment. Functional magnetic resonance imaging data were collected from 11 UD individuals and seven matched O participants during rest. A seed-to-voxel analysis was performed, including the anterior and the posterior cingulate cortex, the bilateral insula, amygdala and hippocampus as seed regions. No group differences in the clinical scales emerged. Compared to O, the UD group showed lower RSFC between the left amygdala and the left cerebellum (lobules VIII), and lower functional coupling between the right hippocampus and the posterior portion of the right middle temporal gyrus. Moreover, UD participants showed higher RSFC between the right amygdala and the anterior cingulate cortex. Our findings suggest RSFC alterations in regions associated with encoding of salient events, emotion processing, memories retrieval and self-referential processing in UD participants, highlighting the potential role of attachment experiences in shaping brain abnormalities also in non-clinical UD individuals.

## 1. Introduction

In his attachment theory, Bowlby [1] assumed the existence of mental representations of interpersonal relationships with attachment figures and their responsiveness in social contexts. Such representations have been labeled “Internal Working Models” (IWMs) and are thought to be stable throughout the lifespan [2,3,4]. IWMs are shaped by early interpersonal experiences with caregivers and are fundamental for social patterns of interactions and general mental health, given their implication in emotion regulation processes [5,6]. Early experiences of neglect and maltreatment have been linked to the development of the unresolved–disorganized (UD) attachment pattern, which is characterized by an incoherent state of mind about childhood attachment memories [7]. UD IWMs contain contradictory expectations to caregivers’ behavior and are related to traumatic memories of early fearful or neglectful experiences [8,9,10]. UD pattern may be considered as a momentary breakdown of an organized strategy to cope with stressful situations: for example, a child may simultaneously show proximity-seeking and avoidant behaviors toward caregivers, which are represented as a source of care and fear at the same time [9,11,12]. Mikulincer and Shaver [13] proposed a three-phase model of attachment system in adults in which the first component involves the monitoring and appraisal of threatening events, the second deals with attachment figure availability and responsiveness, and the third examines the utility of seeking proximity to an attachment figure as a way of coping with threats to safety and well-being. This latter component is thought to be responsible for individual differences in attachment patterns and in corresponding adaptive strategies of emotion regulation: individuals with a UD attachment display emotional dysregulation and dramatic behavioral reactions in response to stressful stimuli [14]. UD pattern appears to be overrepresented in psychiatric patients (43%) and predicts a vulnerability to dissociative disorders [15] and borderline personality disorder [16,17]; nevertheless, individuals with a UD pattern are also found among non-clinical samples (18%) and, although at a lower extent, they display emotional difficulties as well as impulse dyscontrol [7].

In the last few years, neuroimaging studies have highlighted several links between UD attachment and brain irregularities in terms of structural and functional features, with many researches highlighting crucial differences in temporal regions, including the amygdala and hippocampus. In a 30-year longitudinal study, Lyons-Ruth et al. [18] investigated the amygdala volume of 18 low-income participants whose attachment pattern was assessed by employing the Strange Situation Procedure at 18 months of age and who were followed longitudinally to age 29. Authors found increased volume in the left amygdala in participants characterized by maternal and infant components of UD attachment interactions at 18 months of age, and such structural abnormality was also associated with somatic disturbances, brief hallucinatory events, automatisms, visual phenomena, and dissociative episodes. When compared to controls, participants reporting higher exposure to maltreatment and lower levels of maternal care during childhood showed a bilateral enlargement of amygdala volumes; in addition, the severity of maltreatment at 10-11 years of age predicted right amygdala volume in adulthood in longitudinal participants [19]. Buchheim et al. [20] explored the brain activation of participants with an Organized and UD attachment pattern in response to the Adult Attachment Projective, a measure for assessing adult attachment based on narrative responses to a set of eight figures depicting events devoted to activating the attachment system. In this study, participants with a UD attachment with respect to organized ones showed higher activation of medial temporal regions, including the amygdala and the hippocampus, suggesting that the activation of such regions may indicate higher stress in UD individuals resulting from the retrieval of traumatic or negative autobiographical memories during the Adult Attachment Projective. A similar design has been implemented in order to explore the brain activation of the attachment system in healthy participants compared to patients with borderline personality disorder, a clinical population that is often characterized by emotion dysregulation and UD IWMs associated with trauma and abusive experiences in infancy and childhood [21]. Once again, increased amygdala activation was observed in response to the Adult Attachment Projective in participants with UD attachment pattern, regardless of the presence of a borderline personality disorder diagnosis. Nevertheless, unresolved controls, but not borderline patients, displayed further activation in the dorsolateral prefrontal cortex and in the rostral part of the cingulate cortex, suggesting a more functional attempt to cope with negative representations elicited by the Adult Attachment Projective involving emotion regulation, cognitive control and conflict monitoring, with respect to patients who, in turn, appeared overwhelmed and unable to regulate their attachment-related negative states. Blunted amygdala responses have been observed by Kim et al. [22], whoexamined neural activity in mothers classified as having unresolved trauma in the Adult Attachment Interview compared to mothers with no trauma, while observing happy and sad face images of their children. Mothers with no trauma showed higher amygdala activation in response to sad faces of their infants, whereas the opposite pattern was found in traumatized mothers, indicating a possible disengagement mechanism of mothers with unresolved trauma from their infant distress and consequential poor maternal caregiving. Recently, van Hoof et al. [23] hypothesized that atypical amygdala connectivity in UD individuals may representa vulnerability factor for the development of psychopathology; authors examined resting state functional connectivity in 74 adolescents with and without psychiatric disorder that underwent the Adult Attachment Interview in order to assess their current state of mind in relation to attachment memories and found greater functional connectivity between the left amygdala and left lateral occipital cortex, precuneus and superior parietal lobule as well as lower connectivitybetween the left amygdala and medial frontal cortex in UD participants. The authors did not find significant differences in amygdala volume between UD and non-UD adolescents but reported smaller left hippocampal volume and higher functional connectivity between the left hippocampus and the right middle temporal gyrus and the lateral occipital cortex in UD participants [24]. Regarding hippocampal morphology, opposite results have been shown about larger left hippocampal volume in neonates that have been later classified as having a disorganized attachment at 1 year and half of age [25], disorganized children at 10 years of age [26], and adults characterized by maternal withdrawal in infancy and borderline personality disorder features such as self-injury and suicidality [27].

Furthermore, neural activity within the amygdala, medial prefrontal cortex—as a core component of the anterior cingulated cortex—and the hippocampus has been associated with the regulation of several emotional processes, particularly in emotional learning and memory modulation [28]; for example, it has been shown that the inhibition of the dorsolateral prefrontal cortex—which is directly connected to the amygdala—via transcranial magnetic stimulation decreased physiological responding to learned fear, suggesting a pivotal role for this area in a neural network involved in the reconsolidation of fear memories in humans [29]. Similarly, the ventromedial prefrontal cortex appears to have a causal role in fear learning, with patients with a lesion in this region failing in producing physiological adaptive responses during threat acquisition [30]. These findings bring into consideration the therapeutic potential role of non-invasive brain stimulation as an alternative treatment for patients characterized by persistent maladaptive emotional memories, such as those related to attachment [31].

Taken together, evidence suggests that UD individuals may display differences in structural and functional features of the brain when compared with organized subjects at different stages of life, although whether UD adults may have unique patterns of altered resting state functional connectivity is still little characterized. Therefore, the aim of the current study was to examine whether UD attachment is related to specific resting state patterns when compared to individuals with an organized attachment pattern. Participants’ state of mind in relation to their attachment memories was assessed employing the Adult Attachment Interview, which is considered the gold standard measure for attachment patterns in adulthood. We hypothesized that UD individuals would display an altered pattern of resting state functional connectivity with respect to organized participants in specific regions of the brain, including the anterior and the posterior cingulate cortex, the bilateral insula, amygdala and hippocampus. Such regions have shown altered connectivity patterns in UD adolescents [23,24] and are thought to be involved in processes that were found to be impaired in UD individuals such as salient events processing, emotion regulation, memories retrieval and self-referential processing.

## 2. Materials and Methods

### 2.1. Participants

Eighteen individuals (8 female), whose age ranged between 19 and 56 (mean 30.722; SD 10.151) took part in the study. The sample size was similar to other previous studies on non-clinical participants with a UD attachment pattern [12,20,21]. This sample was largely drawn from a larger set of participants involved in a previous study [32]. All participants underwent clinical assessment, including the Adult Attachment Interview (AAI [33]), the Personality Inventory for DSM-5 (PID-5 [34]), and the Symptom Checklist-90-Revised (SCL-90-R [35]); an extended description of the measures as well as additional information about participants selection is provided in Supplementary Material. Using the AAI, 7 individuals (4 female) were classified as UD and 11 (4 female) as organized (O). Groups were matched for age (UD group mean = 35.143, SD = 13.741; O group mean = 27.909, SD = 6.284; Mann–Whitney’s U = 28.000; *p* = 0.375). The authors assert that all procedures contributing to this work comply with the ethical standards of the relevant national and institutional committees on human experimentation. The protocol was approved by the local Ethics Committee of IRCCS Fondazione Santa Lucia in Rome and conformed to the Helsinki Declaration of 1975, as revised in 2008. Informed consent was obtained from all participants included in the study.

### 2.2. Image Acquisition and Analysis

For each participant, we acquired two resting-state functional magnetic resonance imaging (fMRI) scans using an echo planar imaging (EPI) sequence (axial orientation, 72 × 72 matrix, 172 volumes, 38 slices, in-plane resolution = 2.5 × 2.5 mm, slice thickness = 3.6 mm, TR = 2 s, TE = 30 ms, flip angle = 77 deg). During resting-state fMRI scans, participants were asked to lay at rest with eyes closed and not to fall asleep; head movements were minimized with mild restraint and cushioning. MRI scans were collected using a Philips Achieva scanner operating at 3T.

Resting-state data were processed using the CONN toolbox for functional connectivity analysis (v. 19c) (http://www.nitrc.org/projects/conn [36] last accessed 12 July 2021), running on the Statistical Parametric Mapping 12 (SPM12) software (http://www.fil.ion.ucl.ac.uk/spm/ last accessed 12 July 2021). After removal of the initial 4 scans, the functional images were resampled to a voxel size of 2 × 2 × 2 mm^3^, realigned and unwarped; time series were interpolated to correct for slice-timing distortions. ART-based scrubbing [37] for detection of functional outliers was also applied. Structural images were segmented in gray matter, white matter (WM), and cerebrospinal fluid (CSF) for successive use during removal of temporal confounding factors and were normalized to MNI space. Functional data were smoothed using an 8 mm^3^ full-width half-maximum (FWHM) Gaussian kernel.

Temporal confounding factors (i.e., time-courses of WM and CSF BOLD signals, a linear trend, and the six motion parameters derived from the previous realignment procedure) were removed from the BOLD time series of functional data, regressing them out at each voxel. A band-pass filter (0.008 0.09 Hz) was then applied to resulting residual time series.

A seed-to-voxel analysis was performed, including the following theoretically motivated regions as seeds: the anterior and the posterior cingulate cortex, the bilateral insula, amygdala (Figure S1 in Supplementary Materials) and hippocampus. Seeds were selected from the FSL Harvard–Oxford Atlas [38] as implemented in CONN. For each region, and participant seed-based connectivity maps, representing the level of functional connectivity between the seed and every voxel or location in the brain, were computed as the Fisher-transformed bivariate correlation coefficients between the seed BOLD timeseries and each individual voxel BOLD timeseries. Then, the two groups (UD vs. O) were directly compared using two sample ttests. Resulting statistical parametric maps were thresheld using cluster-level FDR-corrected *p* value < 0.05 after forming clusters of adjacent voxels surviving a threshold of *p* < 0.001 uncorrected [39].

## 3. Results

Group clinical information and non-parametrical group comparisons are reported in Table 1. No significant differences between groups emerged in any clinical scale, suggesting that the two groups are comparable in terms of general psychopathological assessment and do not divert from the general population performance [34,35].

**Figure 1:**
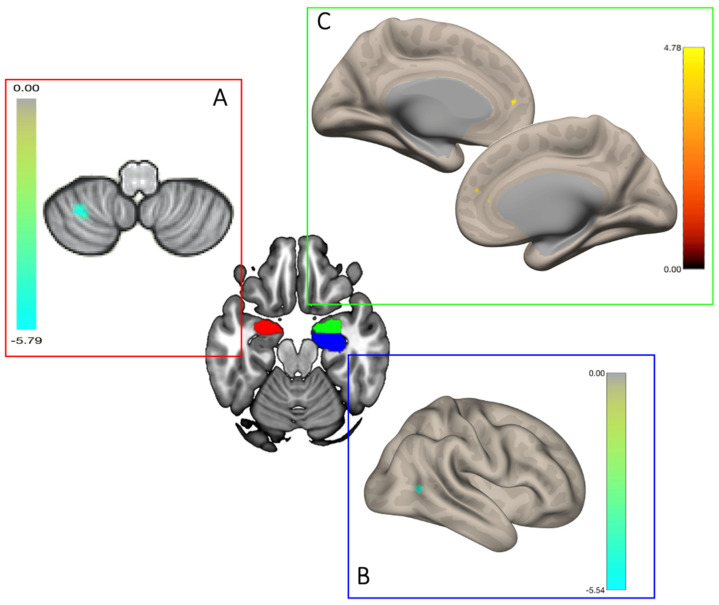
Seed regions are shown in the center, with the left amygdala in red, the right amygdala in green and the right hippocampus in blue. For each region, significant suprathreshold clusters (*p* < 0.05 cluster-size p-FDR) are reported in the panel (**A**–**C**). Clusters of voxels with lower functional connectivity in the UD group are shown in green-to-light blue patches, whereas clusters with higher functional connectivity in the UD group are shown in red-to-yellow patches. **Panel A.** Functional coupling between the left amygdala and the left cerebellum (lobules VIII). **Panel B.** Functional coupling between the right hippocampus and the posterior portion of the right middle temporal gyrus on the lateral surface of the brain. **Panel C.** Functional coupling between the right amygdala and the anterior cingulate cortex on the medial surface of the brain.

RSFC results are summarized in Table 2, along with statistics, cluster size and MNI coordinates for each suprathreshold cluster of between-group differences. Overall, we found that UD and O showed different patterns of seed-to-voxel functional connectivity. Specifically, the UD group showed lower functional coupling between the left amygdala and the left cerebellum (lobules VIII) as compared with the O group (Figure 1A). In addition, the UD group showed lower functional coupling between the right hippocampus and the posterior portion of the right middle temporal gyrus on the lateral surface of the brain (Figure 1B). Otherwise, the UD group showed higher functional coupling between the right amygdala and the anterior cingulate cortex on the medial surface of the brain, as compared with the O group (Figure 1C). Effect sizes for each suprathreshold cluster described above are provided in Figure S2 in supplementary material.

## 4. Discussion

In the current work, we explored resting-state functional connectivity in participants with a UD attachment compared to O individuals, and we found specific patterns of activation in the two groups. Early traumatic experiences of abuse and neglect can lead to the development of a UD attachment pattern that could be further reflected by structural and functional alteration in brain regions associated with encoding of salient events, emotion processing, memories retrieval and self-referential processing. In the current study, we found that UD showed different patterns of functional connectivity in a subset of investigated regions, namely the bilateral amygdala and the right hippocampus, with target voxels that were mainly located in the cerebellum, the anterior cingulate cortex and the posterior middle temporal gyrus. The two groups do not differ in any of the clinical scales assessed, suggesting that differences found in the functional connectivity may be ascribed to the attachment pattern rather than to other confounding clinical variables. This evidence seems to suggest the potential and specific role of traumatic early attachment experiences in resting state functional connectivity specific patterns even in non-clinical UD individuals. For easiness of exposition, the discussion will be divided into subheadings, according to the role of each investigated node.

### 4.1. The Role of the Amygdala: Altered Functional Connectivity of the Bilateral Amygdala in UD Compared to O Participants

The involvement of the amygdala in emotional processes has been showed in many works [40,41,42,43,44,45,46,47], and an analysis of all the functions associated would be beyond the aim of the present study. Therefore, we will focus on aspects more related to the dimension of attachment. From a general perspective, it appears that the intense early stress of childhood maltreatment (such as neglect in caregiving, physical and/or psychological abuse, repeated violence) is associated with long-lasting alterations in the fronto-limbic circuitry [48] and that both amygdala morphology and functioning are associated with several typical behaviors of the UD attachment pattern.

One feature of many adults with UD is substance use. This specific behavior is not necessarily associated with psychopathological pictures but is also associated with subclinical conditions. In a study of drug users of methamphetamine [49], it was found that childhood maltreatment was positively associated with resting state connectivity between the amygdala and several structures including the hippocampus, the parahippocampal gyrus, the inferior temporal gyrus and the orbitofrontal cortex in the right hemisphere, as well as with the cerebellum and the brainstem. With respect to our study, the authors found that connectivity between the amygdala and hippocampus was positively correlated with measures of emotion dysregulation, which is a typical trait of subjects with UD attachment pattern.

Similarly, but from a different perspective, it has been demonstrated that caregiving stressors during infancy were associated with overdeveloped limbic areas [18,25,27,50,51]. With regard to our data, support comes from works by Cortes Hidalgo et al. [26] and Lyons-Ruth et al. [18], who found evidence that a UD in the first 18 months is correlated with enlarged limbic structures years later, suggesting, one more time, the effect of maltreatment on attachment style and amygdala development.

Numerous animal model studies have also reached similar findings. For example, studies in which maternal care and responsiveness were manipulated showed that higher levels of stress due to neglecting maternal caring led to an increased amygdala volume that also persisted at latter developmental stages of life [52,53,54]. This may be due to the features of amygdala developmental trajectory that is characterized by a robust growth during the first few years of life, with a peak around the timing of preadolescence in non-human primates [55] as well as in healthy humans [56], highlighting how early environmental stressors may perturb a typical development in such an area. Similarly, animal and human studies showed that maternal care-related early life stress result in persistent alterations in the amygdala functional circuitry [53], with alterations in functional connectivity of the amygdala with the hippocampus [48,49,57], the medial prefrontal cortex [58] and the anterior cingulate cortex [59,60].

With respect to the functional asymmetries, it has been proposed that the left and right amygdala may play different roles in the processing of attachment stimuli [18] in accordance with the motivational hypothesis of brain asymmetries, which postulates that the left hemisphere is involved in approach-directed behaviors versus the right hemisphere in avoidance-directed behaviors [61]. Accordingly, the left amygdala has been proposed to be preferentially recruited in processing maternal stimuli in childhood [62], whereas the right amygdala appeared to be involved in response to negative or threatening stimuli, with fear circuitry alterations being linked to exposure to childhood maltreatment and neglecting care [63,64]. In the current study, we showed blunted resting-state functional connectivity in both the left and right amygdalae in healthy UD participants, suggesting that the UD attachment pattern may interfere with both approaching and avoiding attitudes. Contradictory expectations to caregivers’ behavior and the displaying of proximity-seeking and avoidance behaviors toward the caregivers at the same time is a typical feature of UD children, which represents their attachment figures as a source of simultaneouscare and fear [8,9,11,12].

Our data seem to support previous studies that link the typical psychological features associated with UD attachment pattern, including emotion dysregulation, anxious and depressive symptoms, as well as dissociative episodes and self-harming behaviors [65,66,67] to alteration ofthe morphology and functioning of this limbic structure [68,69,70,71,72,73]. We may speculate that traumatic memories of early fearful or neglectful experiences found in UD IWMs [8,11] may represent stressful events that interfere with the typical development of amygdala circuitry also in absence of a frank psychopathological picture; in addition, it may be possible that differences in terms of amygdalar resting-state functional connectivity between UD and O individuals may represent one possible brain mechanism associated with psychological distress in UD attachment.

### 4.2. Higher Functional Connectivity between the Right Amygdala and the ACC in UD

The rostral part of the anterior cingulate cortex (ACC) is involved in conflict and error monitoring, decision uncertainty and in monitoring for unfavorable outcomes [74,75,76,77,78,79,80], and it appears to be connected with the amygdala [81,82] in a circuitry devoted to emotion regulation and appraisal [83], with the cingulate cortex exerting an inhibitory influence on the amygdala. Higher functional connectivity between the right amygdala and the rostral part of the ACC may be interpreted as a difficulty in automatic emotion regulation or a lack of cognitive control over emotions [84,85], reflecting the inability of UD individuals in integrating traumatic attachment memories in a coherent and organized representation and their emotional dyscontrol. Consistently, higher activity of the ACC has been previously found in patients who had experienced physical and sexual abuse and developed a post-traumatic stress disorder [86]. However, the anterior cingulate cortex is also part of the so-called Cortical Midline Structures (CMS) system, which has been associated with self-processing not only during stimulus-induced states but also during restingstates characterized by spontaneous thoughts [87,88,89]. Scalabrini et al. [90] proposed a neuropsychodynamic model of personality thattakes into account neural correlates of the self and individuals’ early attachment experiences; such model posits the relational alignment as a prerequisite for the construction of the self, which is described as a neuro-ecological continuum between the brain and the external world and has its origin in the earliest relations with a caregiver. Authors suggest that negative attachment experiences are linked to difficulties in the construction of the self and are reflected in the intrapsychic structure of the individuals. More specifically, in the psychotic personality organization, the relationship between the CMS and the somatosensory network appears to be altered, resulting in a lack of differentiation in processing intrinsic and extrinsic stimuli; in the borderline personality organization, a lack of integration in the brain’s self-other networks, including the CMS, can be observed and may lead to affective and mentalizing dysregulation; and in the neurotic personality organization, difficulties to expand and finalize themselves because their internal conflicts may be reflected in CMS abnormal activity during restingstate as well as during tasks. Moreover, a neural overlap between brain activity during the processing of self-specific stimuli and during restingstate has been specifically found in the ACC [91]. Such rest–self overlap has been described by Northoff [92] as a regional convergence between resting state activity and self-related activity in the CMS system, bringing into consideration the hypothesis that the self can no longer be considered as a higher-order feature of cognitive function but as a basis function of the brain’s spontaneous activity. The ACC appears to play a fundamental role within the CMS in the monitoring of mental states: deficits in the ACC activity have been linked to schizophrenic patients’ impaired internal monitoring of one’s own actions [93] and patients with lesions in the ACC showed apathy, lack of initiative and aberrant social behavior, which could result from an inability to monitor behavioral and mental states [94]. Disorganized, incoherent representations of attachment may prevent important aspects of the self from being integrated, thus predisposing the self-structure to fragmentation and dissociation, with different contradictory self-states (e.g., helpless vs. hostile, victim vs. persecutor)being considered as ensuing from UD attachment [95]. It may be speculated that deficit in the monitoring of self-mental states reflected in abnormal ACC activity may be linked to difficulties in creating a coherent and integrated representation of the self as observed in UD individuals, although further studies are mandatory in order to explore such a hypothesis.

### 4.3. Lower Functional Connectivity between the Left Amygdala and the Left Cerebellum in UD

The amygdala, as well as other medial temporal lobe regions such as the hippocampus, appears to be linked to the cerebellum via ascending projections of the cerebellar fastigial nucleus [96], and resting state functional connectivity has been observed between several cerebellar subregions and the amygdala, indicating the involvement of the cerebellum in emotional processing [97,98]. Clusters of cerebellar activation comparable to cerebellar areas found to be connected to the amygdala in the present study have beenobserved in taste perception [99] and nociception [100,101]. Petrowski et al. [102] found lower brain activity in the left cerebellum/cerebellar vermis in UD when compared to securely attached participants while viewing faces of attachment figures, suggesting a lower ability in UD individuals for emotional perception, bonding, and processing social information. This hypothesis is further supported by our data about lower functional connectivity in healthy UD individuals between the left amygdala and the left cerebellum, more specifically with lobule VIII. To date, the connectivity between the cerebellum and the amygdala has not been extensively studied; our data seem to indicate what other research has already shown, namely the presence of an emotional function in the cerebellum [103,104].

### 4.4. Lower Functional Connectivity between the Right Hippocampus and the Right Middle Temporal Gyrus in UD

The hippocampus has been shown to be sensitive to early life stressors, with smaller hippocampi being found in low-income children [105,106,107] and children exposed to parental separation or loss [108]. From a neurobiological standpoint, the hippocampus is involved in stress-response modulation, providing inhibitory feedback and thus a return of the system to homeostasis [109]. Early life stress has an impact on hippocampal neuroplasticity, with maternal deprivation, neglecting care and infant traumas being correlated to a lower number of available glucocorticoid receptors, resulting in an alteration of the hippocampus-mediated feedback control of the hypothalamus–pituitary–adrenal axis [110,111]. Differences in hippocampal structure as well as in functional activity in response to attachment-related stimuli have been observed in UD compared to O individuals [21,24,25,26], suggesting that the hippocampus may play a role in dysfunctional stress responses-observed UD pattern as well as in the retrieval of unresolved, traumatic or negatively valanced autobiographical material associated with attachment memories. In the current study, we found a significantly lower functional connectivity at rest between the right hippocampus and the right middle temporal gyrus (rMTG) in UD individuals compared to participants with an organized attachment pattern. The right pMTG has been linked to high-level visual processing and in understanding others’ intentions from social cues such as the gaze [112] and fear of social exclusion [113], and functional connectivity between the hippocampus and the rMTG has been associated with the ability of forming new associations during novelty processing [114]. Difficulties in processing social salient stimuli have been observed in UD individuals [32,102] as well as deficits in understanding others’ emotions and mental states in maltreated children [115]. Taken together, this evidence points to altered brain mechanisms underpinning an adequate processing of social stimuli that is reflected in impaired social skills and dysfunctional interpersonal relationships in UD individuals and in their difficulty in forming new, organized representations of self and others. In a recent study, van Hoof et al. [24] found an enhanced connectivity between the hippocampus and the middle temporal gyrus (MTG) at rest in UD participants, which is the opposite trend shown in the current research. One possible explanation regards the inclusion of non-clinical adults in the present study, whereas the sample from van Hoof et al. [24] encompassed adolescents with (i.e., post-traumatic stress disorder, anxiety and/or depressive disorders) and without a psychiatric diagnosis.

## 5. Limitations and Conclusions

One limitation of this study is the small number of participants. Unfortunately, many participants with disorganized attachment refused to participate in the fMRI study or withdrew. Nevertheless, the effect size we observed (Figure S1) supports our results, despite the small sample size. A second consideration is that we did not find any significant difference between groups in any clinical scale, suggesting that the two groups are comparable in terms of general psychopathological assessment and do not divert from the general population performance; if these results may point to a possible role of attachment disorganization in affecting RSFC in absence of a psychiatric diagnosis, it must be considered that our data are not representative of a psychopathological population. Future studies are mandatory in order to further address the specific role of UD attachment in differently affecting RSFC in psychiatric as well as in non-clinical samples.

Limitations notwithstanding, while exploratory, the present work showed differences between UD and O participants in RSFC of brain regions previously linked to disorganized attachment and associated with encoding of salient events, emotion processing, memories retrieval and self-referential processing. Moreover, the majority of the structures that displayed altered RSFC appear to be sensitive to early life stressor and maternal neglect typically observed in UD individuals; in turn, such traumatic early experiences may affect structural and functional features of these regions, including bilateral amygdala, right hippocampus and anterior cingulate cortex. Importantly, our results extend previous knowledge on RSFC and attachment to non-clinical UD individuals, suggesting that differences in brain functional connectivity may occur also in absence of a frank psychopathological picture. Future studies with larger sample sizes are mandatory to generalize results from the present exploratory research and to better address such issues.

## Figures and Tables

**Table 1:** Pre-existing group differences (means and standard deviations) in the clinical scales of the study.

	Organized Attachment (N = 11, 4 F) Mean (SD)	Unresolved/ Disorganized Attachment (N = 7, 4 F) Mean (SD)	Mann-Whitney’s U	*p* Value	Cohen’s *d*
PID-5—Negative Affect	1.024 (0.43)	1.118 (0.65)	35.000	0.751	0.171
PID-5—Detachment	0.504 (0.33)	0.564 (0.78)	46.000	0.497	0.100
PID-5—Antagonism	0.393 (0.31)	0.383 (0.30)	39.000	0.964	0.033
PID-5—Disinhibition	0.688 (0.42)	0.842 (0.61)	31.000	0.497	0.294
PID-5—Psychoticism	0.462 (0.53)	0.900 (0.83)	25.000	0.221	0.629
SCL-90-R—Somatization	46.909 (4.93)	51.714 (9.30)	25.000	0.219	0.646
SCL-90-R—Obsessive-Compulsive	47.000 (8.96)	53.857 (11.98)	23.500	0.173	0.648
SCL-90-R—Interpersonal Sensitivity	48.182 (9.03)	51.857 (12.17)	31.000	0.492	0.343
SCL-90-R—Depression	50.818 (11.64)	54.286 (12.91)	33.500	0.649	0.282
SCL-90-R—Anxiety	52.636 (11.23)	52.143 (10.72)	37.000	0.892	0.045
SCL-90-R—Hostility	49.455 (12.84)	54.143 (11.768)	24.500	0.203	0.381
SCL-90-R—Phobic Anxiety	49.273 (5.62)	53.857 (13.40)	34.000	0.681	0.446
SCL-90-R—Paranoid Ideation	46.636 (10.25)	54.429 (12.05)	23.500	0.173	0.697
SCL-90-R—Psychoticism	46.727 (8.34)	50.429 (10.15)	29.000	0.384	0.399
SCL-90-R—Global Score	49.455 (10.59)	52.571 (12.39)	31.000	0.496	0.270

Note. SD, standard deviation; PID-5, Personality Inventory for DSM-5; SCL-90-R, Symptom Checklist-90-Revised.

**Table 2:** Results of the contrast between the O and UD groups.

Seed/*Target*	x	y	z	Cluster Size	Cluster p-FDR	Cluster p-unc	Peak p-unc	Effect
Amygdala LH								
*Lobule VIII LH*	*−32*	*−56*	*−56*	*64*	*0.018471*	*0.000324*	*0.000028*	−
Amygdala RH								
*ACC*	*2*	*44*	*16*	*57*	*0.037827*	*0.000714*	*0.000066*	*+*
Hippocampus RH								
*pMTG RH*	*54*	*−64*	*6*	*86*	*0.002723*	*0.000066*	*0.000007*	*−*

Note. Target regions are shown in italics. LH, left hemisphere; RH, right hemisphere; ACC, anterior cingulate cortex; pMTG, posterior middle temporalgyrus; +, higher coupling in UD; −, lower coupling in UD.

## Data Availability

The datasets used and/or analyzed for the present paper can be made available upon a reasonable request to the corresponding author.

## Supplementary Materials

The following are available online at https://www.mdpi.com/article/10.3390/brainsci11111539/s1, Participants, Measures, Figure S1: Axial coronal and sagittal views of the left (in red) and the right amygdala (in green), Figure S2: Effect sizes for each suprathreshold cluster with significant between-group differences.
