# Supplementary material for: An Exploratory Study on Resting-State Functional Connectivity in Individuals with Disorganized Attachment: Evidence for Key Regions in Amygdala and Hippocampus
Source: Brain Sci. 2021 Nov 19;11(11):1539. doi: 10.3390/brainsci11111539 (PMC8615787; doi:10.3390/brainsci11111539)
Supplement: Supplementary file 1 [file brainsci-11-01539-s001.zip › brainsci-1423269-supplementary.pdf]

Participants

The original set involved in the previous study was composed of a total of 17 participants classified as having a Unresolved/Disorganized attachment and 46 individuals with an Organized attachment; all of them took part to the behavioral part of the study that has been previously published. Of these, only 12 Unresolved/Disorganized participants agreed to undergo fMRI scan and for this reason, we selected 12 sex- and age-matched Organized participants who agreed to undergo fMRI scan. Of the 12 Unresolved/Disorganized participants, 5 of them refused to complete the fMRI acquisition of resting state functional connectivity due to high level of anxiety and low compliance with the experimental set up. Regarding Organized individuals, 1 participant quitted the fMRI session before completing the resting state functional connectivity recording. Due to these reasons, the final sample of the current study was composed of 7 Unresolved/Disorganized and 11 Organized participants.

Measures

Adult Attachment Interview (AAI [29]). The AAI is a semi-structured clinical interview designed to assess individuals' current state of mind with respect to past caregiver–child attachment-related experiences [11]. The way in which individuals narrate their attachment memories during the AAI (e.g., derogating or minimizing of attachment vs. valuing and rendering a balanced, coherent narrative despite positivity or negativity of actual experience) is supposed to reflect the quality or security of one's current state of mind with respect to attachment. Interpretations of the adult attachment patterns do not rely on the assumption that retrieved memories during the interview represent veridical accounts of early childhood experience; rather, transcripts of the interviews are coded by trained raters according to how coherently people recall their past experiences. Generally, 1 out of 3 possible main classifications is assigned to the most prominent state of mind throughout the interview: secure/autonomous (F), insecure-dismissing (Ds), or insecure-preoccupied (E), of which secure/autonomous is considered the most favorable. A primary classification of an unresolved/disorganized (UD) state of mind may be also assigned when sufficiently marked discussions of experiences of loss, abuse, or other potential trauma are scored for disorientation in reasoning or discourse. In such a case, a secondary (organized) classification (i.e. F, Ds, or E) is assigned for the remaining narrative. When one singular organized state of mind cannot be identified (e.g., because of the presence of marked indications of several states of mind), interviews are coded as cannot classify (CC). The AAIs were transcribed verbatim and were coded by two certified AAI coders according to the AAI coding and classification system [10].

Personality Inventory for DSM-5 (PID-5 [30]). The PID-5 is a 220-item self-report measure of the DSM-5 alternative personality disorder model traits. The PID-5 measures 25 personality traits that can be organized into five overarching domains (i.e., negative affect vs. emotional stability, detachment vs. extraversion, psychoticism vs. lucidity, antagonism vs. agreeableness, and disinhibition vs. conscientiousness). Each trait is assessed by 4 to 14 items and evidence from non-clinical samples indicated that the PID-5 latent trait domain structures were concordant with Five Factor Model traits and demonstrated good convergence with well-established personality trait measures.

Symptom Checklist-90-Revised (SCL-90-R [31]). The SCL-90-R is a self-report questionnaire composed of 90 items exploring the frequency of several psychological symptoms in the last week. Respondents are asked to answer on a 5-point Likert scale ranging from 0 (not at all) to 4 (extremely). The nine clinical subscales are Somatization, Obsessive-Compulsive, Interpersonal Sensitivity, Depression, Anxiety, Phobic Anxiety, Psychoticism, Paranoid Ideation, and Hostility. The global indices include the Global Severity Index (GSI), the Positive Symptom Distress Index (PSDI), and the Positive Symptom Index (PSI). Overall, the SCL-90-R subscales have demonstrated excellent internal consistency (.77 to .90) and test–retest reliability (.78 to .90).

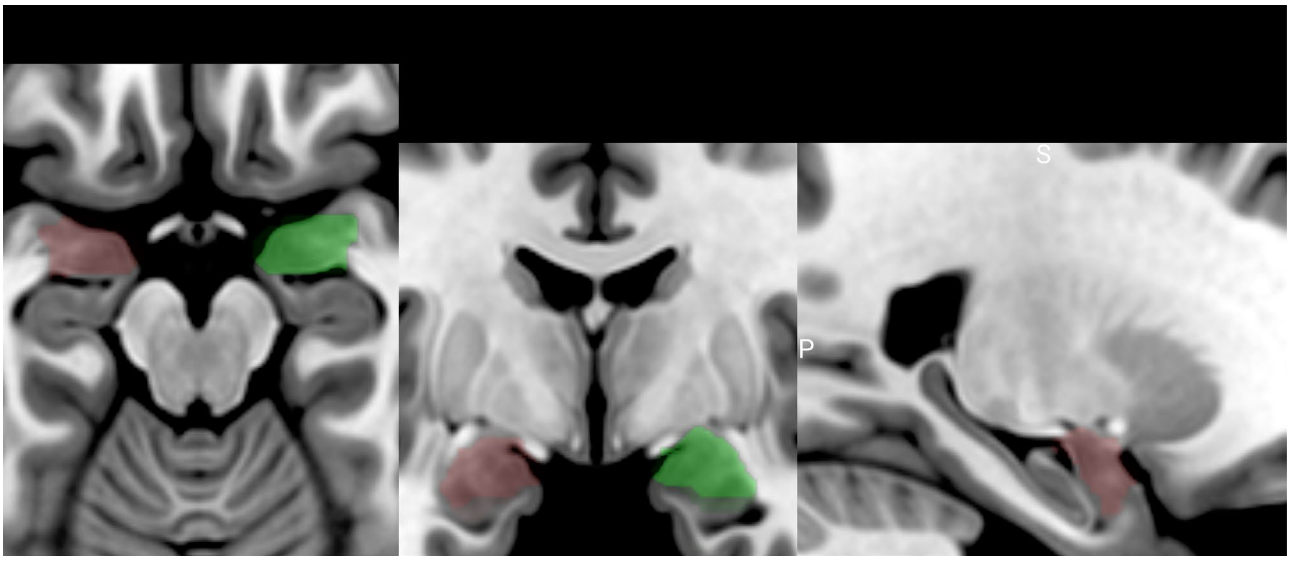

Figure S1. Axial, coronal and sagittal views of the left (in red) and the right amygdala (in green).

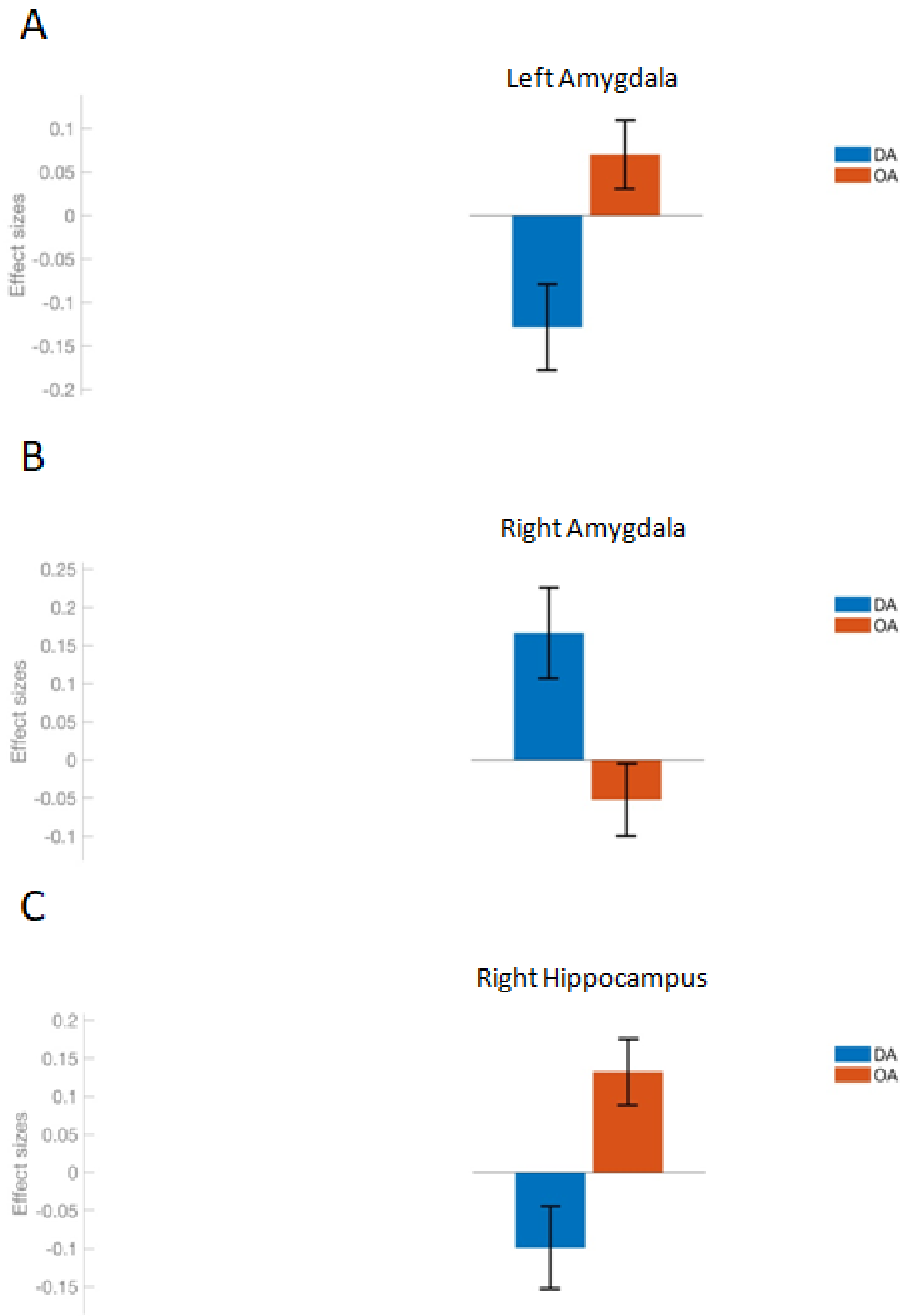

Figure S2. Effect sizes for each suprathreshold cluster with significant between-group differences. The figure shows the between-group effect size for left Amygdala (panel A), right Amygdala (panel B), and right Hippocampus (panel C) respectively. *Note:* DA = Unresolved/Disorganized Attachment; OA = Organized Attachment.
